# 765 Treatment of burns due to application of hair extensions and braiding in younger female patients

**DOI:** 10.1093/jbcr/irac012.318

**Published:** 2022-03-23

**Authors:** Beretta C Coffman, Joan Wilson, Kade Hardy, Farrah Parker, Shana Addison

**Affiliations:** Joseph M. Still Burn Center at Doctors Hospital, Augusta, Georgia; Joseph M. Still Burn Center at Doctors Hospital, Augusta, Georgia; Joseph M. Still Burn Center at Doctors Hospital, Augusta, Georgia; Joseph M. Still Burn Center at Doctors Hospital, Augusta, Georgia; Joseph M. Still Burn Center at Doctors Hospital, Augusta, Georgia

## Abstract

**Introduction:**

Burn injury due to hair styling processes have been reported in the literature, but most of those have been reported to involve flat irons or chemical processing during hair coloring. Moreover, these injuries have predominantly involved adult patients. In the past, our center has seen similar injuries intermittently, with the addition of hot comb burns to the scalp as well.

More recently, a number of younger female patients have been treated for a more uncommon injury related to application of hair extensions. The hair extensions are curled with hot boiling water which is usually taken from the microwave. The ends of the extensions are dipped in the hot water and when they are applied to the hair, the hot water burns the back, shoulders, and scalp.

The purpose of this project is to describe the mechanism of injury from this type of hair styling process and to identify the population at risk. This will serve to raise awareness in the community so that preventive measures might be considered.

**Methods:**

This is a case series describing an unusual mechanism of burn injury reviewing admissions from January 2016 through June 30, 2021.

**Results:**

A total of 35 patients were admitted for this type of burn during this time. 32 were children and 3 were adults. Though this type of injury occurs more commonly in children, it can occur in adults as well. For purposes of this study, the focus will be injuries occurring in children.

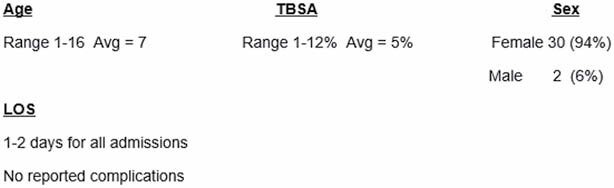

**Conclusions:**

This case series serves as a public service announcement to alert parents of young girls obtaining hair extensions and parents braiding the hair of young children of the potential danger of burn injury from the process. Burn clinicians are obligated to provide education that serves to help prevent such injuries. This presentation serves that purpose as well.

